# Supplementary material for: On the potential sources of a low-frequency sound percept that only a few can perceive
Source: PLoS One. 2026 Mar 27;21(3):e0326818. doi: 10.1371/journal.pone.0326818 (PMC13028376; doi:10.1371/journal.pone.0326818)
Supplement: S7 File — (PDF) [file pone.0326818.s007.pdf]

Fragebogen

1. Wie beurteilen sie die akustische Situation an Ihrem Wohnort auf einer Skala von 1-10?

1	2	3	4	5	6	7	8	9	10
ruhig									laut

2. Wann wurde der Brummton das erste Mal von Ihnen wahrgenommen (MM/JJJJ)?

____

3. Haben Sie zusätzliche, andere akustische Wahrnehmungen unbekannter Herkunft (‚normaler' Tinnitus)?

- ☐ Ja
  Welche:____
- ☐ Nein

4. Ist Ihre Brummtonwahrnehumg eindeutig einem Ohr zuordenbar?

- ☐ Nein
- ☐ Ja, dem linken Ohr
- ☐ Ja, dem rechten Ohr

5. Welche Faktoren verstärken Ihren Brummton?

- ☐ Keine
- ☐ Tieffrequente Schalle
- ☐ Stress
- ☐ Dehydration
- ☐ Schlafmangel
- ☐ Musizieren
- ☐ Medikamente
- ☐ Kopfbewegungen
- ☐ Jahreszeit

- ☐ Externe Schallquellen
- ☐ Flugreisen
- ☐ Sonstige:____

6. Welche Faktoren verringern Ihren Brummton?

- ☐ Keine
- ☐ Tieffrequente Schalle
- ☐ Stress
- ☐ Dehydration
- ☐ Schlafmangel
- ☐ Musizieren
- ☐ Medikamente
- ☐ Kopfbewegungen
- ☐ Jahreszeit
- ☐ Externe Schallquellen
- ☐ Flugreisen
- ☐ Sonstige:____

7. Tritt der Brummton nur an bestimmten Orten auf?

- ☐ Ja
  Welche:____
- ☐ Nein

8. Ist es dort eher leise oder laut? Beurteilen Sie dies auf einer Skala von 1-10:

1 2 3 4 5 6 7 8 9 10
ruhig laut

- ☐ unterschiedlich

9. Tritt der Brummton v.a. zu bestimmten Tageszeiten auf?

- ☐ Ja: morgens
- ☐ Ja: mittags
- ☐ Ja: abends

- Ja: nachts
- Nein

10. Nehmen weitere Familienmitglieder den Brummton wahr?

- Ja
- Nein

11. Wird der Brummton von anderen, nicht verwandten Personen in der gleichen Umgebung mit der gleichen Häufigkeit gehört?

- Ja
- Nein

12. Bestehen bei Ihnen bekannte Erkrankung des Hörsystems oder des Gleichgewichtssystems?

- Ja
- Nein

13. Nehmen Sie regelmäßig Aspirin oder vergleichbare Produkte zur Linderung der Brummtonwahrnehmung ein?

- Ja
- Nein

14. Haben seit der ersten Wahrnehmung des Brummtons Veränderungen hinsichtlich der Lautstärke des Brummtons bemerkt?

- Ja: lauter
- Ja: leiser
- Nein

15. Haben seit der ersten Wahrnehmung des Brummtons Veränderungen hinsichtlich der Tonhöhe des Brummtons bemerkt?

- Ja: tiefer
- Ja: höher
- Nein

16. Haben seit der ersten Wahrnehmung des Brummtons Veränderungen hinsichtlich der Häufigkeit des Brummtons bemerkt?

- ☐ Ja: häufiger
- ☐ Ja: seltener
- ☐ Nein

17. Hat eine Messung am Wohnort das Vorhandensein einer physikalisch nachweisbaren Tieftonschallquelle bestätigt?

- ☐ Ja
- ☐ Nein
- ☐ Nicht vorgenommen

18. Stellt die Wahrnehmung des Brummtons für Sie eine physiologische oder psychologische Belastung dar?

- ☐ Ja
- ☐ Nein

19. Worin vermuten Sie die Ursache für Ihre Wahrnehmung des Brummtons?
Externe Schallquelle

- ☐ Tinnitus
- ☐ Spontane otoakustische Emissionen
- ☐ Sonstiges:____

20. Wie würden Sie die Qualität Ihrer Brummtonwahnehmung beschreiben?

- ☐ Reintonhaft
- ☐ Pulsierend
- ☐ An- und abschwellend
- ☐ Konstant

21. Wie alt sind Sie?

____

22. Welches Geschlecht haben Sie?

- ☐ Männlich
- ☐ Weiblich
- ☐ Divers
